# Morphometric and Biochemical Variation in Seeds of *Agriophyllum squarrosum* (L.) Moq. Across Kazakhstan and Their Implications for Nutritional Quality and Breeding

**DOI:** 10.3390/plants15131937

**Published:** 2026-06-23

**Authors:** Yuliya Genievskaya, Magzhan Almukhamed, Aldabergen Yespanov, Pengshan Zhao, Saule Abugalieva, Yerlan Turuspekov, Alibek Zatybekov

**Affiliations:** 1 Laboratory of Molecular Genetics, Institute of Plant Biology and Biotechnology, Almaty 050040, Kazakhstan; julia.genievskaya@gmail.com (Y.G.); magzanalmuhamed905@gmail.com (M.A.); absaule17@gmail.com (S.A.); yerlant@yahoo.com (Y.T.); 2 Aral Sea Experimental Station of Plant Genetic Resources Named After N.I. Vavilov, a Branch of the South-West Research Institute of Livestock and Plant Growing, Shalkar 031200, Kazakhstan; yespanov60@mail.ru; 3 State Key Laboratory of Ecological Safety and Sustainable Development in Arid Lands, Northwest Institute of Eco-Environment and Resources, Chinese Academy of Sciences, Lanzhou 730000, China; zhaopengshan@lzb.ac.cn

**Keywords:** sand rice, adaptation, amino acid profile, breeding potential, intraspecific trait variation, population differentiation

## Abstract

*Agriophyllum squarrosum* (L.) Moq. (sand rice) is a drought-tolerant psammophytic species with high potential as a climate-resilient food crop due to its nutritional value and adaptation to arid environments. This study evaluates morphometric and biochemical variation in seeds from five natural populations across the deserts of Kazakhstan to assess their breeding potential. Seed morphometric traits showed moderate variability (CVs of 4.71–17.98%), with strong positive correlations among seed length, width, and thousand-seed weight, indicating coordinated development. In contrast, biochemical traits, particularly amino acid composition, exhibited substantially higher variability (CV up to 174.9%), reflecting metabolic flexibility under different environmental conditions. Among the amino acids reliably quantified in this study, histidine was the most abundant, while cysteine, tyrosine, and alanine showed high variability. Total protein content remained relatively stable, reaching up to 34.96% in superior accessions. Multivariate analyses revealed significant population differentiation: Akt1 was the most distinct, whereas Alm1 exhibited superior seed size and mass. Weak correlations between morphometric and biochemical traits suggest their partial independence. Integrated multivariate evaluation identified Akt2 and Alm1 as the most promising populations for breeding. Overall, the observed variation highlights strong potential to select genotypes that combine improved seed size with favorable biochemical characteristics, based on the five amino acids quantified above the LOQ, thereby supporting breeding and domestication efforts.

## 1. Introduction

Climate change and ongoing desertification are among the most critical global environmental challenges, profoundly affecting ecosystem stability, agricultural productivity, and food security [1,2]. Arid and semi-arid regions are particularly vulnerable due to low precipitation, high evapotranspiration, and poor soil fertility [3,4]. In Central Asia, especially in Kazakhstan, deserts and semi-deserts occupy more than 50% of the national territory, making sustainable agriculture extremely challenging [5]. These fragile ecosystems are highly sensitive to climatic fluctuations, and their degradation leads to reduced vegetation cover, increased soil erosion, and declining agricultural outputs [6]. Consequently, there is an urgent need to identify resilient plant species capable of both ecological restoration and agricultural utilization in arid environments.

A prominent representative of these desert ecosystems is *Agriophyllum squarrosum* (L.) Moq., an annual psammophytic species commonly referred to as sand rice, which flourishes across the arid landscapes of Central Asia, northern China, and Mongolia [7,8]. The species is characterized by high levels of environmental plasticity, enabling persistence in habitats defined by hyperaridity, steep thermal gradients, and frequent aeolian sand deposition [9,10]. It functions as a pioneer species on mobile dunes, where its biomass can reduce wind velocity by up to 90%, thereby contributing to sand stabilization and ecosystem restoration [11]. Moreover, its extensive root system, with lateral roots reaching up to 5 m in length, enhances soil structure and nutrient cycling in otherwise infertile sandy soils [8]. These ecological functions position *A. squarrosum* as a key species for combating desertification and restoring degraded landscapes [12].

In addition to its ecological importance, sand rice has gained increasing attention as a potential food crop for arid and marginal lands [13]. Its seeds possess a highly favorable nutritional profile, combining relatively high protein, lipid, and carbohydrate contents. Specifically, sand rice seeds contain approximately 23.2% protein, 9.7% lipids, and up to 45% carbohydrates (dry weight) [14]. Other reports indicate similar values, with about 23% protein, 9% lipids, 45% carbohydrates, 8% crude fiber, and 5% ash, highlighting its balanced composition [15,16]. This nutrient composition is comparable to that of legumes in protein content while maintaining caloric values similar to those of cereals, making sand rice a unique pseudocereal with dual nutritional advantages [17].

Previous studies have reported the presence of numerous essential and non-essential amino acids in *A. squarrosum* seeds, highlighting their nutritional potential [13,14,15]. However, the present study was not designed to provide a comprehensive amino acid characterization and therefore focuses only on amino acids reliably quantified above the analytical LOQ.

Despite these advantageous properties, *A. squarrosum* remains largely undomesticated. Although it has been consumed for over 1300 years in parts of Central Asia and China, its cultivation has not been systematically improved. Several agronomic constraints hinder its domestication, including small seed size (typically 1.3–2.0 mm in length), seed shattering, asynchronous maturation, and variable yield. Under natural conditions, the seed yield of sand rice ranges from 21 to 66 kg/ha [14,18]. Even though breeding efforts have demonstrated that the yield of selected wild sand rice millet in the Tengger Desert can reach 1281 kg/ha [19], these improvements remain limited and inconsistent across environments. Therefore, further research is needed to identify and select superior genotypes with improved morphological and biochemical traits.

Seed morphometric parameters are a critical component of domestication and adaptation. Traits such as seed size, shape, and mass influence dispersal, germination, and seedling establishment, particularly in desert environments where successful recruitment depends on rapid responses to sporadic rainfall events [10]. In *A. squarrosum*, seeds are small, lightweight, and adapted for wind dispersal, reflecting their ecological strategy as a pioneer species. However, significant intraspecific variation in seed morphometric traits has been reported across populations, suggesting high phenotypic plasticity and potential for selection [10]. Understanding this variability is essential for identifying desirable traits for breeding, particularly those associated with increased seed size and yield.

Equally important is the biochemical composition of seeds, which determines their nutritional quality and potential applications. In addition to proteins and lipids, sand rice seeds contain starch (approximately 43.85% of seed weight) and a wide range of bioactive compounds, including phenolic acids and flavonoids [15]. Metabolomic analyses have identified more than 400 metabolites, including amino acids, fatty acids, organic acids, and antioxidants [15]. These compounds contribute not only to nutritional value but also to health-promoting properties, such as antioxidant and anti-inflammatory effects. However, most studies have focused on individual aspects of seed composition, and comprehensive analyses integrating morphometric and biochemical traits remain limited.

In Kazakhstan and Central Asia, this knowledge gap is particularly pronounced. Although the region represents a major part of the natural distribution of *A. squarrosum*, studies on its phenotypic and biochemical diversity remain scarce. Given the environmental heterogeneity of the region—including variations in temperature, precipitation, and soil properties—natural populations are expected to exhibit substantial variation in morphometric and biochemical traits. Recent molecular assessments support this potential: DNA barcoding using ITS and *matK* markers has confirmed species identity while revealing distinct genetic partitions among geographically distant Kazakh populations, including those separated by up to 1300 km in the western and southeastern regions [8]. Furthermore, the development of novel polymorphic SSR markers has facilitated high-resolution interspecific and intraspecific analyses, demonstrating clear segregation between *A. squarrosum* and the sympatric *Agriophyllum minus* Fisch. & C.A. Mey. While current data indicate high interspecific but relatively low intraspecific genetic diversity, the identification of these informative loci provides a critical framework for population structure analysis [20]. Such genetic and biochemical variation represents a valuable resource for selection and breeding, particularly in the development of climate-resilient crops with nutritional profiles—characterized by high lipid and protein content—comparable to legumes and wheat.

Recent studies highlight the need to integrate morphological and biochemical traits to support the domestication of underutilized species such as sand rice. Understanding relationships between seed size and nutrient composition is essential for selecting genotypes with high yield, nutritional quality, and stress tolerance.

This study provides a comprehensive analysis of seed traits in *A. squarrosum*, with particular emphasis placed on seed morphometric traits, amino acid profiles, total protein, and moisture contents. Intraspecific variation and regional adaptation were assessed using natural populations sourced from across Kazakhstan. Significant variability among these populations is expected, and variation in nutritional characteristics was evaluated among populations.

The specific objectives of this study were (i) to characterize the variability of seed morphometric traits in *A. squarrosum* populations from three regions of Kazakhstan; (ii) to determine seed biochemical composition, including total protein, moisture content, and amino acid profiles, and evaluate their nutritional quality; (iii) to investigate the relationships between seed morphometric and biochemical traits; and (iv) to identify promising populations for potential domestication and breeding programs.

## 2. Results

### 2.1. Study Area and Environmental Conditions

**Figure 1:**
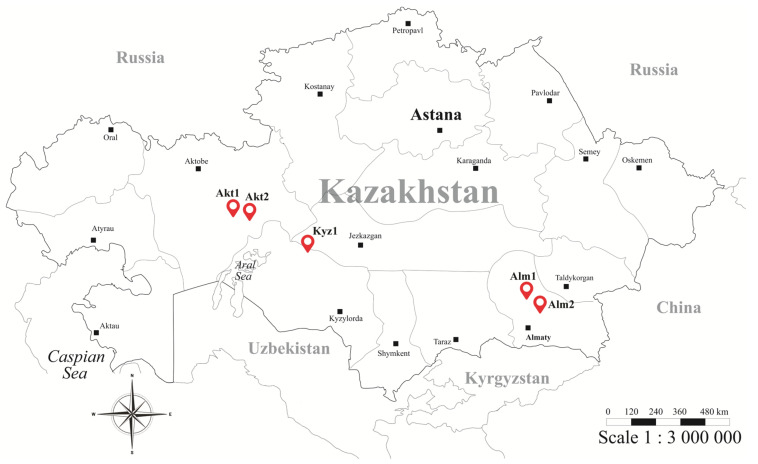
The geographical locations of the collected *A. squarrosum* populations across the Aktobe (Akt1, Akt2), Almaty (Alm1, Alm2), and Kyzylorda (Kyz1) regions.

The sampled populations of *A. squarrosum* were distributed across three regions of Kazakhstan: Aktobe, Almaty, and Kyzylorda. Sampling sites were geographically distinguished, spanning western, southeastern, and southern Kazakhstan, respectively (Figure 1).

In total, 34 individual plant accessions were collected from five populations: 7 from Akt1, 7 from Akt2, 6 from Alm1, 6 from Alm2, and 8 from Kyz1. Climatic conditions were recorded during the 2025 growing season for each population, when the studied plants completed their life cycle (Table 1).

The five *A. squarrosum* sampling sites displayed distinct seasonal patterns in temperature and precipitation. Mean annual temperature (MAT) ranged from +5.1 °C at Akt1 to +9.1 °C at Kyz1, with the coldest conditions in January and the warmest in July. Mean annual precipitation (MAP) varied from 107.5 mm at Kyz1 to 138.4 mm at Alm2, following a bimodal distribution with spring peaks (mainly April–May) and increased moisture in late autumn and winter. The summer months were markedly arid, with the lowest rainfall, especially in July and August.

### 2.2. Seed Morphometric Variation

**Figure 2:**
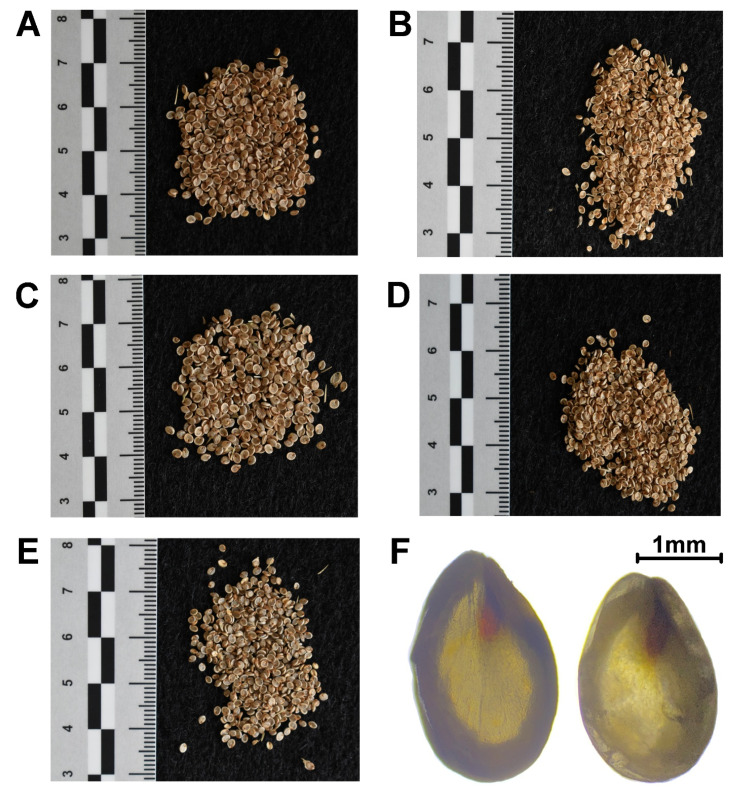
Photo of *A. squarrosum* seeds collected from five populations. (**A**)—Akt1 population, (**B**)—Akt2 population, (**C**)—Alm1 population, (**D**)—Alm2 population, (**E**)—Kyz1 population, (**F**)—micrographs of seeds from the Alm1 population.

Visual inspection of the *A. squarrosum* seed samples across the five study sites revealed a consistent light-brown coloration and a uniform surface texture (Figure 2A–E). The seeds from all populations exhibited the characteristic oval-to-elliptical morphology typical of the species, with a distinct, slightly translucent outer coat and a well-defined internal structure (Figure 2F).

Despite this overall morphological uniformity, seeds of *A. squarrosum* showed pronounced morphometric differentiation in all measured traits (Table 2). Morphometric values presented in Table S1 correspond to accession-level means calculated from measurements of 100–150 seeds per accession.

Across the entire dataset, seed length ranged from 1.87 to 2.67 mm, with a mean of 2.13 mm and moderate variability (CV = 9.84%). Seed width varied from 1.51 to 2.02 mm, with a mean of 1.71 mm and relatively low variability (CV = 7.09%). Seed circularity exhibited the lowest variation among traits (CV = 4.71%), with values ranging from 1.10 to 1.25, indicating a generally stable seed shape. In contrast, thousand-seed weight (TSW) showed the highest variability (CV = 17.98%), ranging from 1.22 to 2.54 g, reflecting substantial differences in seed mass among populations.

**Figure 3:**
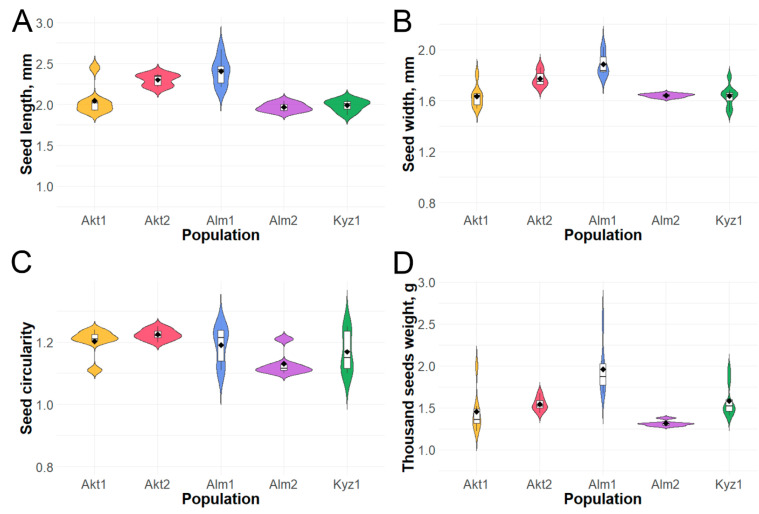
Phenotypic variation in seed morphometric traits across the five populations of *A. squarrosum*. (**A**)—seed length; (**B**)—seed width; (**C**)—seed circularity; (**D**)—thousand-seed weight.

At the population level, seed length ranged from 1.9 to 2.7 mm, with Alm1 showing the greatest median length (2.4 mm) and considerable within-population spread, while Akt1, Alm2, and Kyz1 had the lowest median values (2.0 mm) with moderate dispersion (Figure 3A).

Seed width ranged from 1.5 to 2.0 mm across all populations, with Alm1 displaying the highest median (1.8 mm) and Akt1 the lowest (1.6 mm); all populations showed relatively symmetrical distributions without extreme outliers (Figure 3B). Seed circularity medians were consistently high across populations, particularly in Akt1 and Akt2 (1.2), indicating limited variation in seed shape (Figure 3C). TSW ranged from 1.1 g (Akt1) to 2.7 g (Alm1), with Alm1 displaying both the highest median and the greatest variance, whereas Alm2 showed the most compact distribution (Figure 3D).

**Figure 4:**
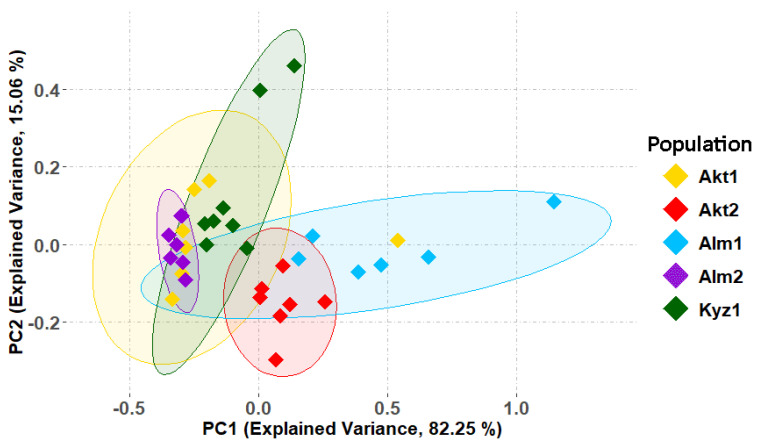
Principal component analysis (PCA) based on seed morphometric traits across five populations of *A. squarrosum*.

Principal component analysis (PCA) of the four morphometric traits revealed that PC1 accounted for 82.25% of the total variance, while PC2 explained an additional 15.06%, together capturing 97.31% of the total variance (Figure 4).

Along PC1, the populations showed clear separation: Alm1 and Akt2 were positioned toward positive values, indicating distinct morphometric profiles compared to the other groups. Conversely, Alm2 and the majority of Akt1 and Kyz1 individuals clustered within the negative PC1 region. Along PC2, most samples remained near the zero axis, though two Kyz1 individuals were distinctly elevated toward the positive extreme, and one Akt2 individual showed a notable negative deviation. Overall, the high cumulative variance explained by the first two axes indicates strong differentiation based on seed morphometric traits. The separation among populations observed in the PCA ordination was further supported by PERMANOVA, which revealed highly significant morphometric differentiation among populations (R^2^ = 0.597, F = 10.735, *p* = 0.001).

### 2.3. Seed Biochemical Variation

The seed biochemical profile of *A. squarrosum* was characterized across five populations, with a focus on moisture content, total protein content, and amino acid composition. Out of the 20 standard amino acids analyzed, five were consistently detected above the quantification limit across samples (Table S2). Descriptive statistics for biochemical traits revealed substantial variation among the components analyzed (Table 3).

Among the amino acids quantified above the analytical LOQ, histidine showed the highest mean concentration (0.134 mg g^−1^ DM), followed by tyrosine (0.048 mg g^−1^), cysteine (0.038 mg g^−1^ DM), alanine (0.023 mg g^−1^ DM), and proline (0.001 mg g^−1^ DM). Variability differed markedly among amino acids, with cysteine (CV = 58.3%) and tyrosine (CV = 43.41%) showing high dispersion, and alanine also exhibiting considerable variability (CV = 41.5%). Histidine was comparatively more stable (CV = 40.23%). Proline displayed extremely high relative variability (CV = 174.9%). However, this value should be interpreted with caution because proline was present at trace or near-detection levels in most samples. Among non-amino acid traits, total protein content showed relatively low variability (CV = 11.29%), whereas moisture content was more variable (CV = 36.86%), indicating differences in seed water status among populations.

**Figure 5:**
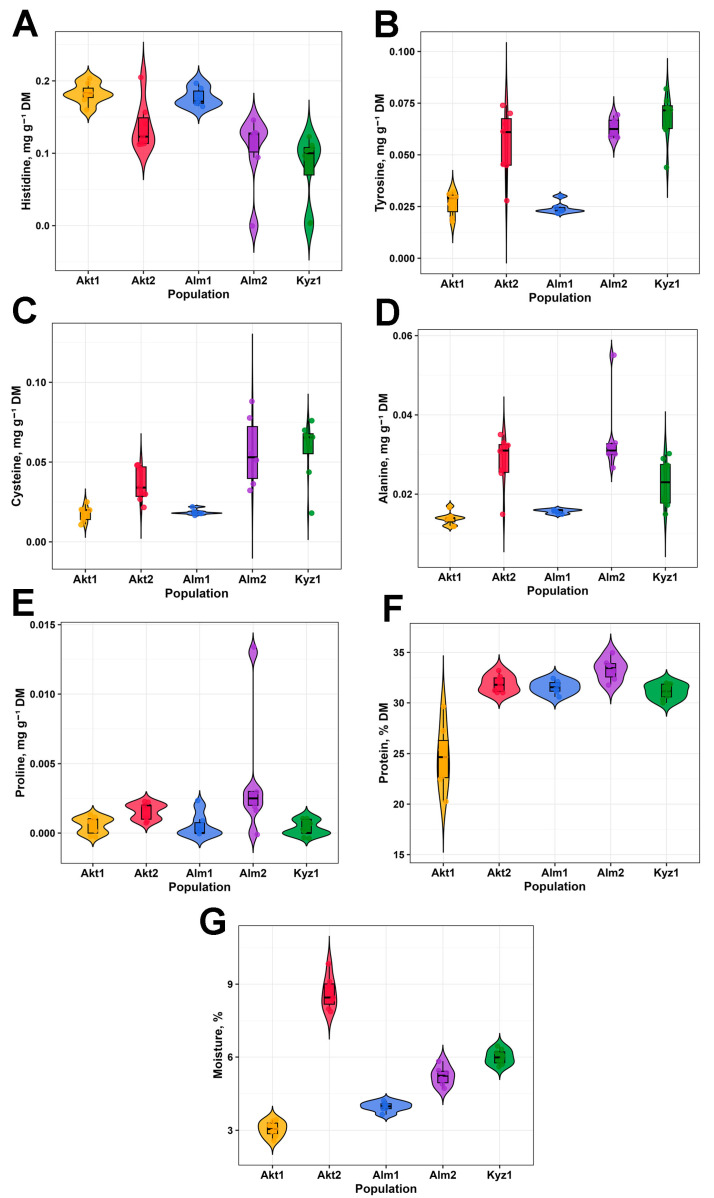
Variation in seed biochemical traits across the five populations of *A. squarrosum*. (**A**)—content of histidine; (**B**)—content of tyrosine; (**C**)—content of cysteine; (**D**)—content of alanine; (**E**)—content of proline; (**F**)—total protein content; (**G**)—moisture content.

At the population level, histidine (Figure 5A) exhibited the highest concentrations, with medians ranging from 0.183 mg g^−1^ DM in Akt1 and Alm1 to 0.177 mg g^−1^ DM mg g^−1^ in Akt1.

Tyrosine (Figure 5B) showed intermediate levels, reaching the highest medians in Kyz1 and Alm2 (>0.06 mg g^−1^ DM) and the lowest in Alm1 (0.02 mg g^−1^ DM). Cysteine (Figure 5C) followed a similar pattern, with the highest median in Kyz1 (0.058 mg g^−1^ DM), whereas Akt1 and Alm1 exhibited the lowest values (<0.020 mg g^−1^ DM). Alanine (Figure 5D) concentrations ranged from 0.014 mg g^−1^ DM in Akt1 to 0.035 mg g^−1^ DM in Alm2, with the greatest variability observed in Akt2 and Alm2. Proline (Figure 5E) occurred at trace concentrations among the detected amino acids (<0.005 mg g^−1^ DM), with values remaining close to the analytical detection threshold in most samples. Alm2 showed the highest median and widest distribution, whereas Akt1, Alm1, and Kyz1 exhibited near-zero values.

Total protein content (Figure 5F) was relatively high in Akt2, Alm1, Alm2, and Kyz1, with median values ranging from 31.121% DM to 33.318% DM, whereas Akt1 exhibited a lower median (24.627% DM) and greater variability (20.26–29.64% DM). Seed moisture content (Figure 5G) varied substantially among populations, from 3.1% in Akt1 to 8.5% in Akt2, with intermediate median values in Alm1 (4.0%), Alm2 (5.2%), and Kyz1 (6.0%), all of which showed relatively low within-population variance. Overall, these results demonstrate pronounced inter-population differentiation in seed biochemical traits, reflecting distinct metabolic profiles among *A. squarrosum* populations.

**Figure 6:**
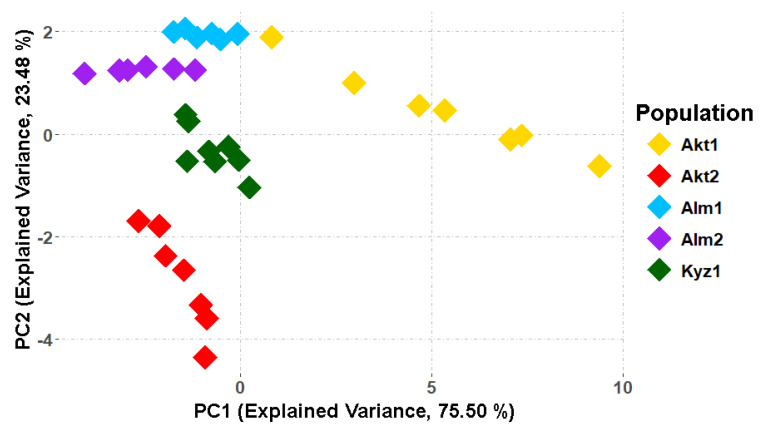
Principal component analysis (PCA) based on the contents of five primary amino acids, total protein, and moisture across five populations of *A. squarrosum*.

PCA of the seven biochemical traits revealed that PC1 explained 75.50% and PC2 23.48% of the total variance, jointly accounting for 98.98% (Figure 6).

Along the PC1 axis, the Akt1 population was strongly separated toward positive values, indicating a distinct biochemical profile compared to the other groups, which predominantly clustered in the negative PC1 region. Along PC2, a clear vertical stratification was observed: Alm1 and Alm2 occupied the most positive scores, while Akt2 was significantly displaced toward negative values, reflecting divergent biochemical compositions. Kyz1 maintained an intermediate position near the PC2 origin. In general, the PCA reveals structured differentiation, with Akt1 and Akt2 representing the most compositionally divergent populations among the *A. squarrosum* germplasm studied.

### 2.4. Trait Relationships and Phenotypic Structure of Populations

**Figure 7:**
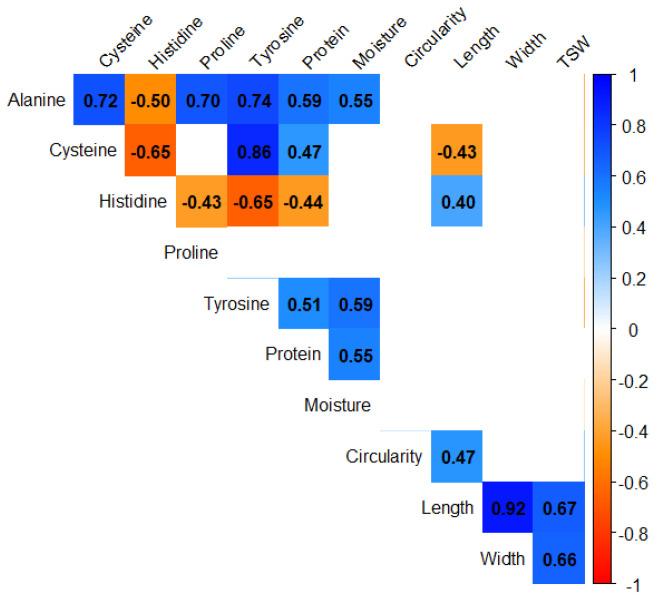
Correlation matrix illustrating the Pearson correlation coefficients (*r*) that remained significant after false discovery rate (FDR) correction for multiple testing (adjusted *p* < 0.05) among seed morphometric and biochemical traits in *A. squarrosum.* TSW—thousand-seed weight.

The phenotypic structure and trait interrelationships among the five *A. squarrosum* populations were characterized through comprehensive correlation and hierarchical clustering analyses. Pearson correlation analysis of seed morphometric and biochemical traits revealed a structured pattern of positive and negative associations (Figure 7).

Among biochemical traits, the strongest positive correlations remaining significant after FDR correction were observed between cysteine and tyrosine (*r* = 0.86), alanine and tyrosine (*r* = 0.74), alanine and cysteine (*r* = 0.72), and alanine and proline (*r* = 0.70), indicating coordinated variation among these amino acids across populations. Histidine was negatively correlated with the other four amino acids (*r* = −0.43 to −0.65) and with protein content (*r* = −0.44). The negative correlations between histidine and other amino acids, as well as protein content, may indicate differential nitrogen allocation among amino acid biosynthetic pathways, potentially reflecting adaptive metabolic regulation under arid environmental conditions. Overall, these patterns suggest coordinated regulation among specific amino acids, in contrast to antagonistic relationships with histidine, indicating distinct metabolic pathways that underlie seed composition.

Among morphometric traits, seed length, seed width, and TSW were strongly positively correlated with each other (*r* = 0.66–0.92), whereas circularity showed a moderate positive correlation only with seed length (*r* = 0.47). When examining cross-category relationships, seed length was negatively correlated with cysteine (*r* = −0.43) but showed a stable, moderate positive correlation with histidine (*r* = 0.40). Protein content showed no significant correlation with any morphometric trait, and seed width showed no significant correlation with any biochemical trait.

**Figure 8:**
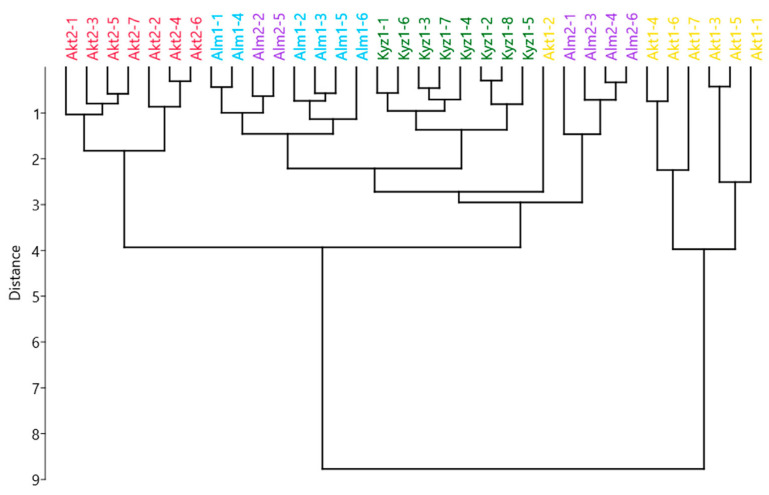
Neighbor-joining tree based on integrated morphometric and biochemical traits demonstrating the hierarchical clustering among *A. squarrosum* accessions of five populations. The colors represent the different populations as follows: yellow—Aktobe1, red—Aktobe2, light blue—Almaty1, purple—Almaty2, and green—Kyzylorda1.

The Neighbor-joining (NJ) tree based on all integrated traits revealed partial phenotypic structuring broadly consistent with population identity, though without complete separation (Figure 8).

Two major hierarchical groupings were observed at a distance of 8.8, at which point the Akt1 population was clearly separated from all other accessions, forming a distinct and well-defined cluster on the root baseline. At a distance of 4.0, the remaining accessions split into two secondary major macro-clusters: one predominantly comprising the Akt2 population on the far left, and another encompassing the Alm1, Alm2, and Kyz1 complex. Further subdivision within the latter complex at a distance of 2.8 revealed the separation of a distinct subgroup, comprising the majority of Alm2 accessions (Alm2-1, Alm2-3, Alm2-4, and Alm2-6). At a distance of 2.3, the Alm1 accessions formed a tight, standalone cluster that embedded the remaining Alm2 outliers (Alm2-2 and Alm2-5), while the Kyz1 population formed a highly homogeneous neighbor group at a distance of 1.5. Overall, Akt1 represents the most phenotypically divergent population in this dataset, whereas Alm1, Alm2, and Kyz1 demonstrate the highest phenotypic similarity, characterized by integrated sub-clustering patterns.

### 2.5. Integrated Multivariate Analysis of Population Differentiation

To comprehensively evaluate phenotypic differentiation, trait–environment relationships, and overall population performance, a series of multivariate and integrative analyses were conducted. Multivariate analysis of variance (MANOVA) revealed highly significant population-level differentiation across all trait categories (Table 4).

The combined dataset showed the strongest statistical separation (Pillai’s Trace = 3.3926, *p* < 2.2 × 10^−16^), followed closely by biochemical traits (Pillai’s Trace = 2.9514, *p* < 2.2 × 10^−16^) and morphometric traits (Pillai’s Trace = 1.5492, *p* = 5.09 × 10^−7^), confirming distinct phenotypic structuring in multivariate space.

**Figure 9:**
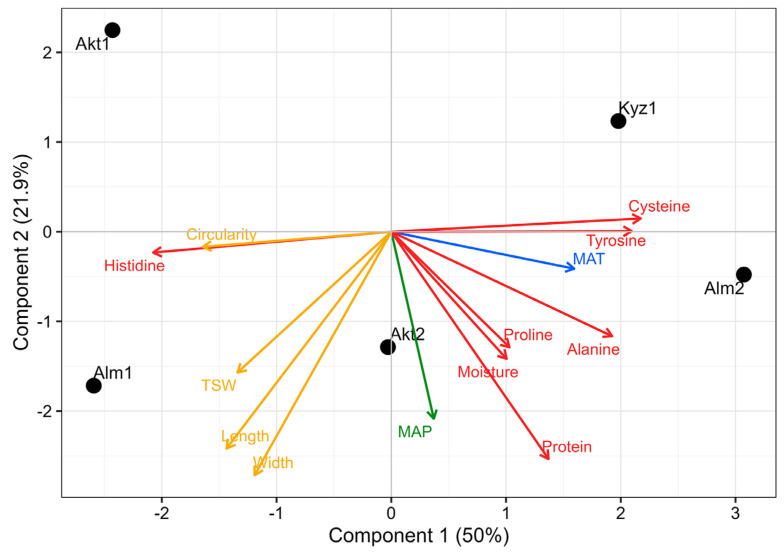
PLS biplot showing the relationship between climatic factors and integrated morphometric (yellow vectors) and biochemical (red vectors) traits across five *A. squarrosum* populations (black circles). MAP—mean annual precipitation (green vector); MAT—mean annual temperature (blue vector).

The relationship between integrated traits and climatic factors was further characterized using a partial least squares (PLS) biplot (Figure 9).

Although the first two components explained 71.9% of the variance in climatic variables (R^2^X) and from 1.6% to 88.9% of the variance in specific phenotypic traits, leave-one-out cross-validation (LOOCV) yielded negative Q^2^ values. This low predictive power is directly attributable to the limited number of environments analyzed (*n* = 5), thereby positioning the PLS framework strictly as a descriptive tool for visualizing climate–trait trends rather than as a predictive model.

Within this ordination space, Component 1 (50%) separated the biochemical and morphometric trait complexes. Specifically, total protein content, moisture, and the majority of amino acids (alanine, proline, cysteine, and tyrosine) loaded positively along Component 1, showing a directional alignment with the climatic drivers (MAP and MAT). Among these, MAT was positioned closer to the cysteine and tyrosine vectors, while MAP displayed a downward orientation towards the Akt2 population. Conversely, histidine and all seed morphometric traits (length, width, TSW, and circularity) loaded negatively along Component 1, demonstrating an antagonistic relationship with both total protein content and the analyzed environmental factors. In terms of population clustering, Alm1 was closely associated with the morphometric trait complex (specifically TSW and length), Alm2 tracked closely with the cysteine/tyrosine cluster, and Akt1 formed an isolated group in the upper-left quadrant, inversely oriented to the primary climatic vectors.

**Figure 10:**
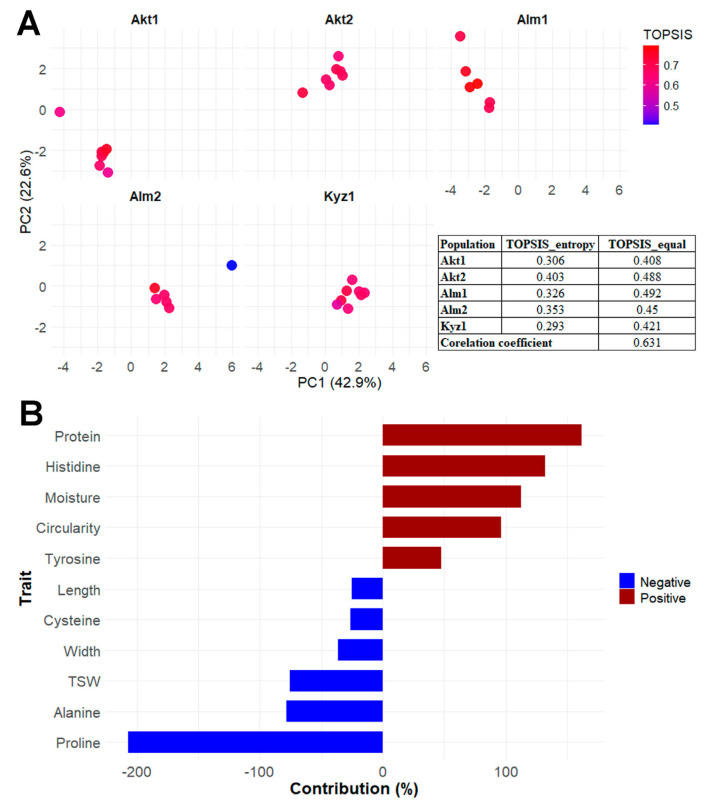
Comparative analysis of *A. squarrosum* populations. (**A**) PCA scores by population with TOPSIS-based evaluation. (**B**) Contribution of individual traits to the multi-criteria evaluation model.

A comparative multi-criteria evaluation combining PCA and the Technique for Order Preference by Similarity to Ideal Solution (TOPSIS) framework revealed structured phenotypic variation and differences in rankings among the studied genotypes (Figure 10).

The first two principal components accounted for 65.5% of the total variance, with PC1 explaining 42.9% and PC2 explaining 22.6% (Figure 10A). In the Alm1 panel, high-scoring genotypes consistently grouped at elevated PC2 values. Conversely, the Alm2 population contained a distinct low-scoring outlier positioned deep in the upper-right quadrant of the ordination space.

The inset table provides a rigorous population-level comparison between entropy-weighted (TOPSIS_entropy) and equal-weighted (TOPSIS_equal) approaches (Figure 10A). For TOPSIS_entropy, the highest integrated value was observed in Akt2 (0.403), followed by Alm2 (0.353) and Alm1 (0.326). Under the TOPSIS_equal approach, Alm1 (0.492) and Akt2 (0.488) exhibited the highest scores. A moderate positive correlation (*r* = 0.631) was observed between the two scoring techniques, indicating partial consistency between weighting schemes while also highlighting differences in the relative contribution of individual traits.

To determine the underlying drivers of this ordination structure, a trait contribution analysis was conducted (Figure 10B). Total protein content emerged as the primary positive driver of the observed phenotypic variance (contributing more than 150%), followed by histidine, moisture content, seed circularity, and tyrosine. Conversely, proline content acted as the dominant negative contributor (exceeding −200%), with alanine, TSW, seed width, cysteine, and seed length also pulling the variance in the negative direction. These results indicate that the top-performing accessions identified by TOPSIS are characterized by a synergistic optimization of high protein/histidine concentration and distinct seed circularity, rather than a mere increase in linear morphometric parameters such as length or width. The difference between entropy-weighted and equal-weighted rankings indicates that population performance is partly influenced by the weighting strategy, although Akt2 and Alm1 consistently ranked among the highest-performing populations.

## 3. Discussion

### 3.1. Coordinated Seed Morphometry and Flexible Biochemical Responses in A. squarrosum Populations

The present study highlights a clear contrast between highly coordinated seed morphometric traits and more flexible biochemical responses in natural populations of *A. squarrosum* across Kazakhstan. Seed morphometric parameters displayed moderate variability, with seed length, width, and TSW showing strong positive intercorrelations (Figure 7). Circularity exhibited the lowest coefficient of variation, reflecting stability in seed shape across populations (Table 2). At the population level, Alm1 consistently produced seeds with the greatest length and width, the highest TSW, and the greatest mass variance, whereas Akt1 tended toward smaller, lighter seeds (Figure 3). These patterns indicate tight developmental integration of size-related traits, consistent with functional requirements related to dispersal, germination, and seedling establishment in sandy dune environments [21].

This coordination aligns well with earlier reports on *A. squarrosum*. It was demonstrated that precipitation and local environmental conditions shape geographic variation in seed size across natural populations, with larger seeds often conferring advantages in rapid seedling recruitment following sporadic rainfall events typical of arid zones [10]. The relatively stable circularity values observed here may reflect stabilizing selection, phenotypic plasticity, or a combination of both mechanisms influencing seed morphology in mobile dune environments, consistent with the species’ pioneer ecology [9,11,22].

In marked contrast, biochemical traits revealed greater flexibility and higher inter-population variability. Among the detected amino acids, cysteine, tyrosine, and alanine showed particularly high coefficients of variation, while total protein content was comparatively more stable, and moisture content displayed substantial fluctuation (Table 3). Strong positive correlations were evident among several amino acid pairs (cysteine/tyrosine, alanine/proline, and alanine/cysteine), indicating coordinated metabolic regulation, whereas histidine exhibited consistent negative correlations with the other amino acids, as well as with total protein and moisture content (Figure 7). One possible explanation is that histidine accumulation reflects differential nitrogen allocation and metabolic partitioning among amino acid biosynthetic pathways. Because histidine biosynthesis is energetically demanding and closely linked to nitrogen metabolism, increased allocation toward histidine production may occur at the expense of other amino acids or storage protein synthesis, particularly under resource-limited arid conditions. Associations between morphometric and biochemical traits remained limited, with only a few moderate negative and positive correlations. Histidine was the only amino acid positively correlated with seed length (*r* = 0.39) and circularity (*r* = 0.35). This moderate association suggests a potential link between nitrogen allocation and seed morphology, though further investigation on larger datasets is required to confirm this trend.

Interpretation of the amino acid composition should be made with caution because only five amino acids were reliably quantified above the analytical LOQ across all samples. Several essential amino acids, including lysine and leucine, could not be consistently quantified and therefore were not included in the comparative analyses. In addition, the analytical protocol was not designed to quantitatively determine hydrolysis-sensitive amino acids. Specifically, performic acid oxidation was not applied prior to hydrolysis, limiting accurate assessment of methionine and cysteine/cystine, while tryptophan was not preserved because alkaline hydrolysis was not performed. Among the identified amino acids, histidine represents an essential component for human nutrition, while cysteine and tyrosine are conditionally essential and play critical roles in antioxidant defense and metabolic regulation [23,24]. The presence of these amino acids suggests that *A. squarrosum* seeds may enhance dietary protein quality, particularly through their roles in key physiological processes. In contrast, alanine and proline, which are non-essential amino acids, are closely associated with central metabolism and stress response. Proline was detected only at trace concentrations with high relative variability, which should be interpreted cautiously as the values were close to the analytical quantification threshold and may partly reflect analytical limitations rather than purely biological stress responses [25,26,27]. The coordinated variation among five amino acids further suggests shared metabolic pathways linked to nitrogen allocation and stress physiology (Figure 7, Table 3). This biochemical flexibility may reflect associations with heterogeneous soil nutrient status, temperature regimes, and water availability across arid zones of Kazakhstan.

The mean total protein content recorded (30.40 ± 0.59% DM) exceeds some previous reports (~23%) [13,14,15] and reaches up to 34.96% DM in superior accessions, reinforcing the species’ favorable nutritional profile as a pseudocereal with protein levels comparable to legumes. The relatively high mean protein content observed in this study compared with previous reports (~23%) may reflect the combined effects of population-specific adaptation, environmental conditions during the sampling year, and methodological differences among studies. In particular, NIR-based estimates calibrated with PLS regression may yield slightly different protein estimates than traditional Kjeldahl approaches commonly used in earlier investigations. Because protein content was estimated using NIR spectroscopy rather than direct chemical determination, the reported values should primarily be interpreted comparatively within the present dataset and not assumed to be directly equivalent to values obtained using alternative analytical methodologies. However, the absence or low detection of several essential amino acids limits a comprehensive assessment of protein quality. The limited detection of amino acids should be interpreted with caution. Acid hydrolysis is known to cause extensive degradation of tryptophan, while quantitative determination of sulfur-containing amino acids generally requires prior oxidation with performic acid. Because neither alkaline hydrolysis to preserve tryptophan nor performic acid oxidation was included in the analytical workflow, the present dataset does not provide a complete quantitative amino acid profile. Therefore, the present nutritional assessment should be considered preliminary, as a complete profile of essential amino acids could not be obtained. Future studies employing complementary analytical approaches, including targeted determination of hydrolysis-sensitive amino acids, will be necessary to provide a more comprehensive evaluation of protein quality and amino acid balance in *A. squarrosum* seeds. Therefore, while *A. squarrosum* seeds have potential as a source of functionally important amino acids; their nutritional value would benefit from further evaluation, particularly regarding amino acid balance and complementarity with other dietary protein sources.

PCA underscored these differential patterns. For morphometric traits, the first component captured most of the variance and clearly separated populations, particularly highlighting Alm1 and Akt2 (Figure 4). Although the relatively small number of accessions limits the robustness of inferences from ordination-based analyses, the PCA patterns were consistent with the significant population differentiation detected by PERMANOVA, supporting the biological relevance of the observed morphometric structure. Biochemical PCA showed even stronger population structuring, with Akt1 distinctly separated along the primary axis (Figure 6). Such results indicate that while seed morphology remains relatively canalized to preserve core ecological functions in dune habitats, biochemical composition, especially amino acid profiles, offers greater plasticity for local optimization. This decoupling of structural and compositional traits is a common strategy in desert annuals, enabling persistence under variable conditions without compromising dispersal morphology [7,10].

These contrasting patterns between morphometric stability and biochemical flexibility provide a mechanistic basis for the population-level differentiation observed across environmental gradients.

### 3.2. Phenotypic Differentiation and Population Structure Across Environmental Gradients

Populations of *A. squarrosum* displayed clear yet incomplete phenotypic differentiation across the three ecological regions of Kazakhstan. MANOVA revealed highly significant differences among the five populations across morphometric and biochemical traits, as well as in the integrated dataset (Table 4). These differences were associated with environmental gradients in temperature and precipitation, with western Aktobe sites experiencing cooler conditions and southern/southeastern sites showing warmer temperatures and varying moisture regimes (Table 1).

The NJ tree constructed from all integrated phenotypic traits revealed a hierarchical structure consistent with geographic and climatic positioning (Figure 8). At the highest distance level, Akt2 formed a distinct, compact cluster separate from all other accessions. Akt1 constituted a secondary major cluster, while Alm1, Alm2, and Kyz1 showed closer phenotypic affinity with noticeable overlap among accessions. Morphometric differentiation was prominent, with Alm1 characterized by superior seed size and mass (Figure 3), and biochemical differentiation was particularly strong, again isolating Alm2 (Figure 6) and showing population-specific patterns in five amino acid contents (Figure 5).

This incomplete differentiation, characterized by well-defined population clusters alongside partial overlap, suggests a dynamic balance between local adaptation to contrasting arid environments and possible ongoing gene flow [28]. Earlier molecular investigations using ITS and *matK* barcoding, as well as SSR markers, identified genetic partitions between western and southeastern populations of *A. squarrosum* in Kazakhstan, separated by large distances, yet reported relatively low overall intraspecific genetic diversity, indicating historical connectivity and effective dispersal mechanisms [8,20]. The current phenotypic data complement these findings, revealing structured trait variation associated with environmental gradients, with no evidence of complete isolation.

Comparable patterns of partial differentiation have been documented in other Central Asian desert and psammophytic species, where strong selective pressures along aridity and temperature gradients are moderated by wind-mediated seed dispersal and the dynamic nature of dune habitats [7,10,29]. In *A. squarrosum*, the pronounced divergence of the western Akt1 population may be associated with its cooler and potentially more variable climatic regime, resulting in distinct biochemical optima. Conversely, the closer clustering of Almaty (southeastern) and Kyzylorda (southern) populations suggests shared ecological constraints in warmer dune systems, with similar phenotypic patterns despite geographic separation.

The substantial within-population variation and overlap observed in the NJ analysis further underscore the role of phenotypic plasticity, a key trait that enables this annual pioneer species to colonize and persist across heterogeneous sandy substrates [9,11]. Such plasticity, combined with gene flow, likely buffers populations against extreme environmental fluctuations characteristic of Central Asian deserts. However, without common garden or reciprocal transplant experiments, the relative contributions of genetic differentiation and environmental plasticity to the observed phenotypic variation cannot be disentangled.

Phenotypic differentiation in *A. squarrosum* is biologically meaningful and structured by environmental gradients across Kazakhstan, with western populations representing a phenotypically distinct group and southeastern/southern populations showing greater similarity. This incomplete structuring, maintained by connectivity, preserves valuable intraspecific diversity for both conservation and utilization.

### 3.3. Climate–Trait Relationships and Ecological Trade-Offs

PLS regression analysis revealed structured, axis-specific associations between climatic variables (MAP and MAT) and seed morphometric and biochemical traits (Figure 9). In contrast to preliminary expectations, total protein content, moisture, and the majority of amino acids (alanine, proline, cysteine, and tyrosine) loaded positively along Component 1, aligning directionally with both MAP and MAT. Conversely, histidine and all seed morphometric parameters (length, width, TSW, and circularity) were tightly clustered in the opposite quadrant (loading negatively on Component 1), indicating a clear antagonistic relationship with the climate-driven biochemical traits.

These climate–trait associations are consistent with the hypothesis of ecological trade-offs in resource allocation under varying stress regimes [30,31]. The divergent orientation between seed size metrics and the climate–biochemical complex indicates that under conditions of shifting precipitation (MAP) and temperature (MAT), allocation priorities may differ among populations. Specifically, the positioning of the Alm1 population near the TSW and length vectors suggests that localized environmental factors favor structural seed investment. In contrast, the Akt2 and Alm2 populations align more closely with intensive allocation of nitrogen and moisture. This high variability in functional trait responses reflects flexible metabolic adjustments crucial for coping with pronounced seasonal aridity and severe summer droughts [10,32]. The distinct isolation of Akt1 and its inverse relationship to primary climatic drivers further highlights that population-specific genetic backgrounds or microenvironmental factors can override macroclimatic trends in regulating seed phenotypic profiles.

Pearson correlation analysis further illuminated these trade-offs. Strong positive associations were observed among four amino acids, in contrast to the antagonistic positioning of histidine relative to other amino acids, protein, and moisture (Figure 7). Morphometric traits were tightly integrated with one another but showed only limited direct correlations with biochemical parameters, suggesting that dispersal-related morphology and nutritional composition can be optimized to some extent independently. The distinct biochemical profile of Akt1 (Figure 6) may be associated with the cooler western environment and may differ from profiles at warmer southern and southeastern sites.

Such patterns are consistent with observations in other psammophytes and desert annuals of Central Asia and northern China, where local climatic conditions have been associated with variation in drought tolerance, temperature response, and nutrient storage traits related to drought tolerance, temperature response, and nutrient storage, supporting the species’ role as an effective pioneer on mobile dunes [7,13,14,33]. The documented plasticity enables *A. squarrosum* to maintain reproductive success and population persistence across wide environmental gradients.

Thus, climatic gradients were associated with trait variation and ecological trade-offs observed among *A. squarrosum* populations. This responsiveness enhances the species’ ecological amplitude and underscores its value as a model for understanding adaptation in arid ecosystems under ongoing climate change.

### 3.4. Implications for Nutritional Value and Breeding of Sand Rice

This study demonstrates substantial population-level variation in seed protein content and the concentrations of the amino acids reliably quantified across populations of *A. squarrosum* in Kazakhstan. However, because only a subset of amino acids could be consistently quantified, conclusions regarding overall nutritional quality and amino acid balance should be considered preliminary (Table 2 and Table 3). Protein levels reached up to 34.96% DM in superior accessions, while variation in the five quantified amino acids demonstrates substantial biochemical diversity among populations. The substantial intraspecific variation in both morphometric and biochemical traits provides a rich genetic resource for improvement.

Although larger seeds are advantageous for breeding due to improved seedling vigor, nutritional reserves, and handling efficiency, excessive selection toward increased seed size could potentially reduce dispersal efficiency and colonization capacity in mobile dune systems. Therefore, future domestication strategies should balance agronomic improvement with maintenance of the species’ ecological role in sand stabilization and pioneer succession.

Multi-criteria evaluation revealed that population rankings depended on the weighting scheme applied. Under entropy weighting, Akt2 achieved the highest overall score, whereas Alm1 ranked highest under equal weighting. Despite these differences, both populations consistently ranked among the top-performing groups and therefore represent promising material for breeding and domestication programs (Figure 10). These accessions combine larger seed size and mass with competitive biochemical profiles, making them particularly promising parental materials for breeding and domestication programs [34].

From a breeding perspective, the observed patterns facilitate simultaneous selection for key agronomic traits, such as increased seed size and TSW (to improve yield and mechanical handling), while maintaining or enhancing protein content and specific amino acid balances. The partial decoupling between morphometric and biochemical trait sets reduces potential trade-offs and allows targeted improvement [35]. The documented population structure (Figure 8) offers a framework for strategic germplasm utilization—incorporating germplasm from divergent western populations (Akt1) and high-performing southeastern material (Alm2)—while preserving local adaptation.

Ecologically, superior genotypes could be deployed to enhance sand stabilization and ecosystem restoration efforts in desertification-prone areas of Central Asia, building upon the species’ natural role as a pioneer that reduces wind velocity and improves soil structure [11,12]. Nutritionally, sand rice holds promise as a climate-resilient crop that can contribute to food security in arid and semi-arid regions facing increasing environmental pressures [2,13,36]. The present study should be regarded as an exploratory assessment of phenotypic and biochemical variability in *A. squarrosum*. The relatively small number of accessions per population limits statistical power. Therefore, the observed patterns should be confirmed in larger-scale studies. Future research directions should prioritize integrating genomic tools, building on existing SSR markers and DNA barcoding [20], alongside common garden experiments and reciprocal transplants to partition genetic and plastic components of trait variation. Multi-year and multi-site field trials under varying climatic conditions, combined with broader metabolomic and lipid profiling and comprehensive amino acid analysis incorporating performic acid oxidation and alkaline hydrolysis for tryptophan determination, will provide deeper insights into nutritional quality and genotype-by-environment interactions. Development of marker-assisted selection strategies informed by climate–trait associations (Figure 9) will accelerate domestication progress.

Overall, the comprehensive phenotypic diversity documented in this study firmly establishes *A. squarrosum* as a promising species for simultaneous ecological restoration and sustainable nutritional production in Central Asia. Targeted breeding, leveraging the identified superior populations and trait relationships, can help unlock its full potential amid continuing desertification and climate challenges.

## 4. Materials and Methods

### 4.1. Plant Material and Sampling

Five natural populations of *A. squarrosum* were sampled across three ecologically distinct regions of Kazakhstan—Aktobe (Akt1, Akt2), Almaty (Alm1, Alm2), and Kyzylorda (Kyz1)—in 2025. These regions represent western, southeastern, and southern arid zones of Kazakhstan, characterized by sandy desert soils typical for the natural habitat of *A. squarrosum*. Geographic coordinates of sampling sites were recorded using a handheld GPS device Garmin eTrex 10 (Garmin Corporation, New Taipei City, Taiwan).

At each site, mature seeds were collected from randomly selected individual plants per population to capture within-population variability, with each plant considered a biological replicate. A total of 34 individual plants (accessions) were sampled across the five populations, including 7 accessions from Akt1, 7 from Akt2, 6 from Alm1, 6 from Alm2, and 8 from Kyz1. To further minimize the probability of sampling closely related individuals, mature plants were collected at a minimum distance of 20 m from one another whenever local population density permitted. Following collection, the seeds were thoroughly air-dried, cleaned to remove the husks, and stored in paper bags at room temperature until further analysis.

Climatic parameters were summarized to describe environmental conditions at each sampling location. Monthly temperature (°C) and precipitation (mm) data for five sampling sites were obtained from publicly available climatic databases [37]. Since *A. squarrosum* is an annual species with a single-season life cycle, climatic data from 2025 were used to reflect the actual environmental conditions during the plants’ growth and seed development.

### 4.2. Morphometry and Biochemical Analysis of Seeds

Seed morphometric traits were quantified using the MARViN ProLine seed analyzer (MARViTECH GmbH, Wittenburg, Germany) through high-resolution digital imaging and automated image analysis software. For each accession (individual plant), 100–150 mature seeds were randomly selected and scanned under standardized conditions to measure the mean length (mm), width (mm), and circularity [(seed perimeter^2^)/(4π × area)]. Thousand-seed weight (TSW, g) was determined for each accession.

Moisture (%) and crude protein (%) content in seeds were determined using an Infralum FT-12 NIR analyzer (Lumex, Saint Petersburg, Russia) in accordance with ISO 12099:2017 [38]. Cleaned samples (50 g) were scanned in triplicate over the 800–2500 nm range, and spectral data were processed using Spectralab software (Lumex, Saint Petersburg, Russia) with PLS regression. Protein values were estimated using NIR spectroscopy calibrated with PLS regression and therefore represent indirect analytical estimates rather than direct determinations obtained by wet-chemistry methods such as Kjeldahl analysis. Total protein content was subsequently expressed on a dry-matter basis using the measured moisture content for each accession. Micrographs of seeds were obtained with Levenhuk MED D10T LCD (Levenhuk Inc., Moscow, Russia).

### 4.3. Amino Acid Profiling

Amino acid profiling was conducted using High-Performance Liquid Chromatography (HPLC). All analyses were performed in triplicate to ensure analytical reproducibility. HPLC-grade methanol, acetonitrile, isopropanol, phenylisothiocyanate (PITC), trimethylamine (TEA), sodium acetate, and amino acid standards (LAA21) were obtained from Sigma-Aldrich (St. Louis, MO, USA). Standard stock solutions of 20 amino acids (1 mg/mL) were diluted to five concentration levels ranging from 0.01 to 1.0 mg/mL to construct calibration curves, which demonstrated high linearity (R^2^ > 0.990). Limits of detection (LOD) and quantification (LOQ) were determined for each amino acid based on signal-to-noise ratios of 3 and 10, respectively. Method precision was evaluated through repeated injections of standards, yielding relative standard deviations (RSD) below 5%. L-nor leucine was used as an internal standard.

Seed samples were ground into a homogeneous fine powder using mill IKA A10 (IKA-Werke GmbH & Co, Staufen, Germany) and subsequently defatted with n-hexane in a Soxhlet apparatus for 8 h to eliminate lipid interference. Total protein hydrolysis was carried out following the AOAC 982.30 protocol [39]. A 100 mg aliquot of the defatted sample was hydrolyzed in 10 mL of 6 M HCl supplemented with 0.1% phenol. To prevent oxidative degradation of labile amino acids, hydrolysis tubes were purged with nitrogen (N_2_) and vacuum-sealed prior to incubation at 110 ± 1 °C for 24 h. After hydrolysis, samples were filtered through a 0.22 µm PTFE membrane, evaporated to dryness under vacuum at 50 °C, and reconstituted in 0.1 M HCl.

It should be noted that acid hydrolysis with 6 M HCl may partially degrade certain amino acids, particularly tryptophan, and sulfur-containing amino acids may also be partially affected despite the protective measures employed. Tryptophan is largely degraded under acidic hydrolysis conditions, whereas accurate quantification of sulfur-containing amino acids (methionine and cysteine/cystine) typically requires prior performic acid oxidation, which was not performed in the present study. Furthermore, several amino acids were present at concentrations below the analytical limit of quantification (LOQ), preventing their reliable determination across all samples. Consequently, only amino acids consistently detected above the LOQ were included in subsequent statistical analyses.

Amino acids were derivatized to PITC derivatives using the Pico-Tag method. The derivatization reagent consisted of PITC, TEA, and isopropanol in a 1:1:8 (*v*/*v*/*v*) ratio. An aliquot of 100 µL of each sample was dried, reacted with 20 µL of the derivatization reagent for 20 min at room temperature, and subsequently vacuum-dried to remove excess reagents. The resulting PTC derivatives were reconstituted in the initial mobile phase prior to analysis.

**Figure 11:**
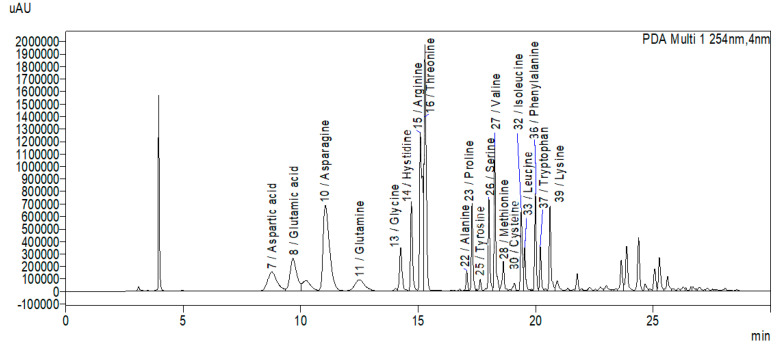
High-Performance liquid chromatography (HPLC) profile of 20 amino acid standards, showing chromatographic separation and peak identification at 254 nm.

Chromatographic separation was performed using a Shimadzu Nexera LC-40D XR system equipped with an SPD-M40 photodiode array (PDA) detector (Shimadzu Corporation, Kyoto, Japan). Separation was achieved on a Supelco Discovery C18 column (250 mm × 4.6 mm, 5 µm) (Sigma-Aldrich/Merck, Bellefonte, PA, USA) maintained at 40 °C. The mobile phase consisted of acetonitrile (99.9%) with 0.1% acetic acid (mobile phase A) and deionized water (99.9%) containing 0.1% acetic acid and 0.1 M sodium acetate (mobile phase B). A gradient elution program was applied at a flow rate of 1.0 mL/min as follows: 0.0–7.5 min, 5% A/95% B; 7.5–20.0 min, linear gradient from 5% to 50% A; 20.0–25.0 min, linear gradient from 50% to 80% A; and 25.0–30.0 min, re-equilibration from 80% to 5% A. Detection was carried out at 254 nm with spectral confirmation in 200–400 nm (Figure 11). Amino acid and protein concentrations were expressed on a dry matter basis (mg g^−1^ DM) using the measured moisture content for each accession.

### 4.4. Multivariate and Multi-Criteria Analysis of Phenotypic Traits

Statistical analyses were performed to evaluate variation and relationships among seed morphometric and biochemical traits. Basic descriptive statistics, including minimum, maximum, mean, standard error (SE), standard deviation (SD), variance, and coefficient of variation (CV), were calculated for all measured traits.

The PCA was performed separately for two groups of traits: seed morphometry (length, width, circularity, and TSW) and biochemical traits (amino acids, total protein, and moisture content). Because of the relatively small sample size, PCA was used primarily as an exploratory ordination method to visualize phenotypic structure rather than for formal inference. To further evaluate population differentiation, a permutational multivariate analysis of variance (PERMANOVA) based on Euclidean distances and 999 permutations was performed. Pairwise relationships among traits were evaluated using Pearson’s correlation coefficients (*r* at *p* < 0.05). To account for multiple testing, *p*-values obtained from Pearson correlation analyses were adjusted using the FDR procedure, and only correlations with adjusted *p* < 0.05 were considered significant. Phenotypic relationships among individual samples were further explored using the NJ clustering approach based on Euclidean distance matrices derived from integrated phenotypic traits in PAST4 [40].

MANOVA was performed to test for significant differences among populations based on seed morphometry and biochemical traits. Assumptions of multivariate normality and homogeneity of covariance matrices were evaluated prior to analysis. Multivariate significance was assessed using Pillai’s Trace statistic [41].

A PLS regression analysis was performed to evaluate the relationships between climatic variables and seed traits. MAP and MAT were used as predictor variables, while morphometric and biochemical seed traits were used as response variables. Variables were mean-centered and unit variance scaled prior to analysis. Model performance was evaluated using R^2^X, R^2^Y, and Q^2^ statistics. The PLS model was fitted with two components and validated using leave-one-out cross-validation (LOOCV) to assess the strength of climate–trait associations.

TOPSIS was used to evaluate the overall performance of accessions across all traits. The traits were treated as benefit criteria, except moisture content, which was considered a cost criterion. The decision matrix was normalized using min–max normalization to remove scale effects. Two weighting schemes were applied. In the entropy-weighted approach (TOPSIS_entropy), criterion weights were calculated according to Shannon entropy theory [42], allowing objective weighting based on trait variability and information content. In the equal-weighted approach (TOPSIS_equal), all traits were assigned identical weights. For both approaches, ideal and anti-ideal solutions were defined, Euclidean distances from each accession to these reference points were calculated, and a relative closeness coefficient was obtained as the final TOPSIS score. Accessions were ranked according to this coefficient, with higher values indicating superior overall performance. Rankings obtained under the two weighting schemes were subsequently compared to assess the influence of trait weighting on genotype evaluation.

All analyses and plots were carried out in R v. 4.6.0 [43] using the packages igraph, corrplot, vegan, stats, biotools, candisc, pls, dplyr, and ggplot2.

## 5. Conclusions

Natural populations of *A. squarrosum* across three regions of Kazakhstan exhibit substantial variation in seed morphometric and biochemical traits, highlighting their value for breeding and domestication. Morphometric traits were relatively stable and strongly coordinated, reflecting functional constraints associated with wind dispersal and seedling establishment in arid dune environments. Seed circularity was particularly conserved among populations, suggesting stabilizing selection for an optimal seed form. In contrast, biochemical traits, especially amino acid composition, showed considerably greater variability, indicating population-specific metabolic responses to differences in climate, soil conditions, and water availability. The observed decoupling between structural and compositional traits is advantageous for breeding because it enables the simultaneous improvement of seed size and nutritional quality without strong phenotypic trade-offs. Multivariate analyses revealed significant population differentiation along geographic and climatic gradients. Among the studied populations, Alm1 and Akt2 consistently ranked among the highest-performing groups in multi-criteria evaluations, making them promising genetic resources for future breeding programs. The elevated protein content observed in superior accessions (up to 34.96%), together with the presence of several nutritionally important amino acids, supports the potential of *A. squarrosum* as a nutrient-rich pseudocereal. However, because a complete essential amino acid profile could not be established, the nutritional assessment remains preliminary. Future studies incorporating comprehensive metabolomic analyses and optimized quantification of sulfur-containing amino acids and tryptophan are needed to fully assess protein quality and breeding potential. Overall, integrating morphometric and biochemical traits provides a robust framework for selecting elite genotypes adapted to harsh environments and for supporting sustainable agriculture and food security in Central Asia.

## Figures and Tables

**Table 1:** Mean monthly temperature and precipitation profiles (2025) for the sampling sites of *A. squarrosum* populations.

Month	Temperature, °C					Precipitation, mm				
	Akt1	Akt2	Alm1	Alm2	Kyz1	Akt1	Akt2	Alm1	Alm2	Kyz1
January	−15.8	−14.4	−14.5	−13.8	−10.7	10.8	12.4	13.0	12.0	12.0
February	−14.5	−13.1	−12.0	−11.2	−9.4	9.3	11.1	11.0	10.1	11.0
March	−6.5	−5.1	−4.5	−3.8	0.0	12.4	14.2	9.0	8.4	15.0
April	+7.7	+8.7	+9.5	+10.2	+11.8	16.2	15.1	19.9	20.0	12.5
May	+16.7	+18.1	+17.8	+18.5	+19.7	14.1	12.9	25.3	26.0	5.5
June	+22.4	+23.4	+23.8	+24.6	+25.8	5.6	7.2	1.0	8.0	5.0
July	+24.4	+25.6	+25.8	+26.5	+27.7	6.0	1.2	2.0	5.3	1.3
August	+22.6	+23.6	+23.8	+24.3	+25.7	0.6	0.2	5.3	6.5	5.8
September	+14.8	+16.0	+17.5	+18.1	+18.1	1.6	3.2	4.0	4.3	2.0
October	+5.3	+6.7	+9.8	+10.4	+9.1	16.4	18.0	10.5	11.7	12.4
November	−4.1	−2.7	−2.0	−1.2	−0.6	11.3	13.3	15.0	14.0	13.0
December	−11.4	−10.0	−10.8	−10.1	−7.9	12.0	13.6	12.0	12.1	12.0
MAT	+5.1	+6.4	+7.0	+7.7	+9.1	–				
MAP	–					116.3	122.4	128	138.4	107.5

MAT—mean annual temperature; MAP—mean annual precipitation.

**Table 2:** Descriptive statistics of seed morphometric traits across the studied *A. squarrosum* populations.

Trait	Min	Max	Mean	SE	SD	CV, %
Seed length, mm	1.87	2.67	2.13	0.04	0.21	9.84
Seed width, mm	1.51	2.02	1.71	0.02	0.12	7.09
Seed circularity	1.10	1.25	1.18	0.01	0.06	4.71
TSW, g	1.22	2.54	1.57	0.05	0.28	17.98

SE—standard error; SD—standard deviation; CV—coefficient of variation; TSW—thousand-seed weight.

**Table 3:** Descriptive statistics of seed biochemical traits expressed on a dry matter basis across the studied *A. squarrosum* populations.

Trait	Min	Max	Mean	SE	SD	CV, %
Alanine content, mg g^−1^ DM	0.012	0.055	0.023	0.002	0.009	41.50
Cysteine content, mg g^−1^ DM	0.011	0.088	0.038	0.004	0.022	58.30
Histidine content, mg g^−1^ DM	0.000	0.205	0.134	0.009	0.054	40.23
Proline content, mg g^−1^ DM	0.000	0.013	0.001	0.000	0.002	174.90
Tyrosine content, mg g^−1^ DM	0.018	0.082	0.048	0.004	0.021	43.41
Total protein content, % DM	20.26	34.96	30.40	0.59	3.43	11.29
Moisture content, %	2.58	9.83	5.44	0.34	2.01	36.86

SE—standard error; SD—standard deviation; CV—coefficient of variation; DM—dry matter.

**Table 4:** MANOVA results of population-based differentiation across morphometric, biochemical, and combined trait categories.

**Morphometric Traits**						
	Df	Pillai’s Trace	Approx. *F*	Num Df	Den Df	*p*
Population	4	1.5492	4.5827	16	116	5.09 × 10^−7^
Residuals	29					
**Biochemical Traits**						
	Df	Pillai’s Trace	Approx. *F*	Num Df	Den Df	*p*
Population	4	2.9514	10.454	28	104	<2.2 × 10^−16^
Residuals	29					
**Combined Data**						
	Df	Pillai’s Trace	Approx. *F*	Num Df	Den Df	*p*
Population	4	3.3926	11.171	44	88	<2.2 × 10^−16^
Residuals	29					

Df—degree of freedom; Approx. *F*—Approximate F-statistic; Num Df—Numerator degrees of freedom; Den Df—Denominator degrees of freedom.

## Data Availability

All data generated or analyzed during this study are included in this published article and its Supplementary Materials.

## Supplementary Materials

The following supporting information can be downloaded at: https://www.mdpi.com/article/10.3390/plants15131937/s1, Table S1: Accession-level seed morphometric traits and biochemical characteristics expressed on a dry matter basis; Table S2: Raw data on amino acid composition of *A. squarrosum* seeds.

## Abbreviations

The following abbreviations are used in this manuscript:

CV	Coefficient of variation
HPLC	High-Performance liquid chromatography
MANOVA	Multivariate analysis of variance
MAP	Mean annual precipitation
MAT	Mean annual temperature
PLS	Partial least squares
TOPSIS	Technique for order preference by similarity to ideal solution
TSW	Thousand-seed weight
